# VACCINATION STATUS OF PATIENTS WITH CHRONIC LIVER DISEASE FOLLOWED UP AT A REFERENCE CENTER IN BRAZIL

**DOI:** 10.1590/S0004-2803.24612026-023

**Published:** 2026-08-24

**Authors:** Maria Elizabete Calore NEIVA, Maria Lucia Gomes FERRAZ, Raimundo Araújo GAMA, Antônio Eduardo Benedito SILVA

**Affiliations:** 1 Universidade Federal de São Paulo (UNIFESP), Disciplina de Gastroenterologia, Mestrado em Gastroenterologia, São Paulo, Brasil.; 2 Universidade Federal de São Paulo (UNIFESP), Disciplina de Gastroenterologia, São Paulo, Brasil.

**Keywords:** Chronic liver disease, vaccination coverage, Immunization, hepatitis vaccines, Doença hepática crônica, cobertura vacinal, imunização, vacinas contra hepatites

## Abstract

**Background:**

Patients with chronic liver disease (CLD) often experience varying degrees of immune dysfunction, making them vulnerable to multiple preventable infectious diseases through appropriate vaccination.

**Objective:**

To evaluate vaccination status and vaccine coverage among patients with chronic liver disease (CLD) receiving care at a tertiary referral center.

**Methods:**

This cross-sectional observational study was conducted at the Hepatology Outpatient Clinic of Hospital São Paulo, Escola Paulista de Medicina, Federal University of São Paulo (UNIFESP), São Paulo, Brazil. A convenience sample of adult patients with different etiologies of CLD was prospectively included. Participants underwent a standardized interview, review of vaccination records, and serological testing for hepatitis A and hepatitis B.

**Results:**

Among 104 patients (61.5% women, median age 51 years), chronic hepatitis C (63.5%) and hepatitis B (10.6%) were the most common causes of CLD. Advanced liver fibrosis was identified in 46.1% of the patients. Vaccination records were available for 82.7% of participants, but only 45.1% showed serological evidence of hepatitis B immunization (anti-HBs positive). Positive anti-HAV IgG serology was detected in 75% of patients, whereas only 14.4% had documented hepatitis A vaccination. Vaccination coverage for other vaccines was as follows: meningococcal conjugate vaccine (8.7%), pneumococcal vaccine PPSV23 (25%), influenza vaccine (53.8%), tetanus-diphtheria vaccine (60.6%), measles-mumps-rubella vaccine (23%), yellow fever vaccine (28.8%), and COVID-19 vaccination coverage was 92.3%.

**Conclusion:**

Vaccination coverage among patients with chronic liver disease remained lower than expected for several recommended vaccines, even in a specialized referral center. These findings highlight the importance of systematic assessment of immunization status and reinforcement of vaccination strategies in patients with chronic liver disease.

## HIGHLIGHTS

• This study aimed to evaluate vaccination coverage among patients with chronic liver disease followed at a tertiary reference center in Brazil.

• An observational analytical study was conducted with 104 consecutive patients, including vaccination card review and hepatitis A and B serological assessment.

• Immunization rates were low for several recommended vaccines, including hepatitis B (45.1% anti-HBs positive), pneumococcal (25% up to date), and meningococcal (8.7%).

• These findings demonstrate underutilization of immunobiological agents and highlight the need for improved medical guidance and structured vaccination strategies in this high-risk population.

## RESUMO

**Contexto:**

Pacientes com doença hepática crônica (DHC) frequentemente apresentam diferentes graus de disfunção imunológica, tornando-os vulneráveis a diversas doenças infecciosas preveníveis por meio da vacinação adequada.

**Objetivo:**

Avaliar o estado vacinal e a cobertura vacinal de pacientes com doença hepática crônica (DHC) acompanhados em um centro terciário de referência.

**Métodos:**

Estudo observacional transversal realizado no Ambulatório de Hepatologia do Hospital São Paulo, Escola Paulista de Medicina, Universidade Federal de São Paulo (UNIFESP), São Paulo, Brasil. Foi incluída prospectivamente uma amostra de conveniência composta por pacientes adultos com diferentes etiologias de DHC. Os participantes foram submetidos a entrevista padronizada, revisão dos registros vacinais e testes sorológicos para hepatites A e B.

**Resultados:**

Foram avaliados 104 pacientes (61,5% mulheres; mediana de idade de 51 anos). As causas mais frequentes de DHC foram hepatite C crônica (63,5%) e hepatite B crônica (10,6%). Fibrose hepática avançada foi identificada em 46,1% dos pacientes. Registros vacinais estavam disponíveis para 82,7% dos participantes, porém apenas 45,1% apresentavam evidência sorológica de imunização contra hepatite B (anti-HBs positivo). Sorologia positiva para IgG anti-HAV foi observada em 75% dos pacientes, enquanto apenas 14,4% possuíam registro de vacinação contra hepatite A. A cobertura vacinal para as demais vacinas foi a seguinte: vacina meningocócica conjugada (8,7%), vacina pneumocócica polissacarídica 23-valente (PPSV23) (25%), vacina contra influenza (53,8%), vacina dupla adulto contra tétano e difteria (dT) (60,6%), vacina tríplice viral (sarampo, caxumba e rubéola) (23%), vacina contra febre amarela (28,8%) e vacina contra COVID-19 (92,3%).

**Conclusão:**

A cobertura vacinal entre pacientes com doença hepática crônica permaneceu inferior ao esperado para diversas vacinas recomendadas, mesmo em um centro especializado de referência. Esses achados reforçam a importância da avaliação sistemática do estado vacinal e do fortalecimento das estratégias de vacinação em pacientes com doença hepática crônica.

## INTRODUCTION

Chronic liver diseases (CLD) have a high global prevalence, with varying etiologies depending on the geographical region. Regardless of the cause, CLD can lead to severe complications such as cirrhosis and hepatocellular carcinoma, often resulting in liver transplantation or death. Although the definition of CLD lacks consensus, it is undeniable that disease progression impairs both innate and adaptive immunity, thereby increasing the risk of infections, particularly in cases of advanced cirrhosis^1^ ^-^ ^3^.

The relationship between the degree of fibrosis in CLD and immune dysfunction can be attributed to the liver’s role in regulating immune homeostasis, among other functions^3^ ^-^ ^5^. Immune dysfunction is observed regardless of the underlying liver disease etiology, but it appears to be more pronounced in alcohol-related liver diseases^5^. Consequently, vaccination plays a crucial role in the management of CLD, considering the disease’s pathophysiology and predisposition to preventable infections. In patients with advanced liver disease, the immune response tends to impair antibody seroconversion. Therefore, vaccination should be administered as early as possible during the disease course to maximize effectiveness^6^ ^-^ ^9^.

In Brazil, specialized immunobiological agents are available for patients with chronic diseases through the Reference Centers for Special Immunobiologicals (RCSIs or CRIEs in Portuguese). The RCSI guidelines specifically recommend influenza, hepatitis A, hepatitis B, pneumococcus, and meningococcus C vaccines for patients with CLD. In 2019, the updated guidelines included additional vaccines such as Td (tetanus and diphtheria adsorbed vaccine for adults), triple viral (mumps, rubella, and measles - MRM), yellow fever vaccine and COVID-19. Furthermore, the conjugate vaccine against meningococcus ACWY was recommended for specific situations involving patients with CLD^10^.

## OBJECTIVE

To evaluate vaccination status and vaccine coverage among patients with chronic liver disease (CLD) receiving care at a tertiary referral center.

## METHODS

This cross-sectional observational study was conducted at the Hepatology Outpatient Clinic of Hospital São Paulo, Escola Paulista de Medicina, Federal University of São Paulo (UNIFESP), São Paulo, Brazil.

A convenience sample of 104 adult patients with chronic liver disease (CLD) and different disease etiologies was prospectively included.

Participants underwent a standardized interview, review of vaccination records, and serological testing for hepatitis A and hepatitis B. The interview assessed the presence of vaccination records, adherence to recommended vaccines, methods of vaccine record-keeping, and self-reported medical counseling regarding immunization.

The vaccines analyzed included hepatitis A, hepatitis B, pneumococcal, meningococcal, influenza, tetanus-diphtheria (Td), measles-mumps-rubella (MMR), yellow fever, and COVID-19 vaccines.

Participants without vaccination records were classified as having undocumented vaccination status, except for hepatitis A and hepatitis B, which were additionally evaluated through serological testing. All participants underwent testing for anti-HAV IgG, HBsAg, anti-HBs, and total anti-HBc.

The study was approved by the Ethics Committee of the Federal University of São Paulo (approval number 1036/2017), and all participants provided written informed consent.

### Statistical analysis

Because this was primarily a descriptive study, no inferential comparative analyses were performed. Continuous variables were described using median and range, while categorical variables were expressed as absolute numbers and percentages. Statistical analyses were performed using IBM SPSS Statistics version 24 (IBM Corp., Armonk, NY, USA).

## RESULTS

### Baseline characteristics

A total of 104 patients were included in the study. The median age was 51 years (range: 18-60), and 61.5% were women. Only 10 participants (9.6%) had completed higher education.

The most common underlying liver disease was chronic hepatitis C (63.5%), followed by hepatitis B (10.6%), nonalcoholic steatohepatitis (7.7%), primary biliary cholangitis (7.7%), alcohol-related liver disease (3.8%), autoimmune hepatitis (3.8%), and schistosomiasis (1.9%). One patient had Wilson disease.

Regarding liver fibrosis, 52% of patients had mild fibrosis (F0-F2), while 46.1% had advanced fibrosis (F3-F4). Fibrosis staging was primarily assessed by liver biopsy (54.8%), followed by transient elastography (FibroScan**®) (34.6%) and clinical evidence of cirrhosis (6.7%).**

Among the 36 cirrhotic patients, 34 (94.4%) were classified as Child-Pugh class A, while one patient each was classified as class B and class C.

Ten participants (9.6%) were receiving immunosuppressive therapy. Table 1 summarizes the baseline characteristics of the study population.

**TABLE 1:** Baseline characteristics of the 104 included patients.

Characteristics	n=104
Age, years	51 (18-60)
**Female gender, n (%)**	64 (61.5)
**Liver disease, n (%)**	
HCV	66 (63.5)
HBV	11 (10.6)
NASH	8 (7.7)
Primary biliary cholangitis	8 (7.7)
Alcohol	4 (3.8)
Autoimmune hepatitis	4 (3.8)
Schistosomiasis	2 (1.9)
Wilson disease	1 (1.0)
**Liver fibrosis, n (%)**	
F0-F1	28 (26.9)
F2	24 (23.1)
F3	12 (11.5)
F4	36 (34.6)
Not evaluated	4 (3.8)
**Diagnosis of fibrosis, n (%)**	
Liver biopsy	57 (54.8)
Transient elastography	36 (34.6)
Clinical evidence of cirrhosis	7 (6.7)
Not evaluated	4 (3.8)
**Child-Pugh classification, n (%)**	
A	34 (94.4)
B	1 (2.8)
C	1 (2.8)
**Use of immunosuppressors**	10 (9.6)

### Analysis of the vaccination card

A total of 86 patients (82.7%) presented vaccination records during the interview. Only 14.4% had documented hepatitis A vaccination. Regarding hepatitis B vaccination, 55 patients (52.9%) had received three doses, four (3.8%) had received four doses, and ten (9.6%) had completed two full vaccination schedules. Thirty-three patients (31.7%) had no documented hepatitis B vaccination; however, nine had chronic hepatitis B and six had evidence of previous HBV infection.

Regarding pneumococcal vaccination (PPSV23), 69 participants (66.3%) had no vaccination record, while 26 (25%) were considered adequately vaccinated according to the study criteria.

For influenza vaccination, 56 participants (53.8%) had documentation of vaccination during the most recent campaign. Meningococcal vaccination coverage was low, with only nine patients (8.7%) presenting vaccine records.

Concerning tetanus-diphtheria (Td) vaccination, 63 participants (60.6%) were considered adequately vaccinated. MMR vaccination records were identified in 24 patients (23%), while yellow fever vaccination was documented in 30 (28.8%).

Regarding COVID-19 vaccination, eight patients (7.69%) had not received any vaccine dose.

Table 2 summarizes these findings.

**TABLE 2:** Analysis of the vaccination card of the 104 included patients.

	n=104
**Vaccination card, n (%)**	
Not available	18 (17.3)
Available, brought to the interview	86 (82.7)
**Hepatitis A vaccine, n (%)**	
No record	89 (85.6)
1 dose	5 (4.8)
2 doses	10 (9.6)
Hepatitis B vaccine, n (%)	
No record	33 (31.7)
1 dose	1 (1.0)
2 doses	1 (1.0)
3 doses	55 (52.9)
4 doses	4 (3.8)
6 doses	10 (9.6)
**PN 23 vaccine, n (%)**	
No record	69 (66.3)
1 dose > 5 years	9 (8.7)
1 dose < 5 years	11 (10.6)
2 doses	15 (14.4)
**Influenza (flu) vaccine, n (%)**	
No record	38 (36.5)
Last campaign	18 (17.3)
Previous campaigns, except the last	10 (9.6)
Last and previous campaigns	38 (36.5)
**Meningococcal C vaccine, n (%)**	
No record	95 (91.3)
Record	9 (8.7)
**dT or Td Vaccine , n (%)**	
No record	31 (29.8)
1 dose	3 (2.9)
2 doses	1 (1.0)
3 doses < 10 years	41 (39.4)
Booster dose > 10 years	6 (5.8)
Booster dose < 10 years	22 (21.2)
**Triple viral vaccine, n (%)**	
No record	80 (76.9)
1 dose	20 (19.2)
2 doses	4 (3.8)
**Yellow fever vaccine, n (%)**	
No record	74 (71.2)
Record	30 (28.8)
**Covid 19 Vaccine, n (%)**	
No record	8 (7.69)
1 - 3 doses	43 (41.34)
>= 4 doses	53 (50.96)

Categorical variables are reported as number (percentage). PN: pneumococcus. dT or Td: diphtheria and tetanus.

### Viral hepatitis A and B serological profiles

Hepatitis A and B serological profiles were obtained for all participants. Positive anti-HAV IgG serology was identified in 78 patients (75%). Because only 14.4% had documented hepatitis A vaccination, previous HAV exposure or undocumented previous vaccination may explain most cases of seropositivity. Regarding hepatitis B, isolated anti-HBs positivity, suggesting previous vaccination, was detected in 47 participants (45.1%).

Ten patients had records of two complete hepatitis B vaccination schedules (six doses), of whom four were anti-HBs positive. In addition, anti-HBs positivity was identified in four patients without documented vaccination records, suggesting previous immunization with loss of vaccination documentation.

Table 3 summarizes the hepatitis A and B serological profiles.

**TABLE 3:** Hepatitis A and B virus serological profile of the 104 included patients.

Serological status, n (%)	n=104
anti-HAV IgG +	78 (75)
anti-HAV IgG -	26 (25)
HBsAg +/anti HBc IgG +/ anti HBs -	11 (10.6)
anti-HBs + (alone)	47 (45.1)
anti-HBc IgG + / anti HBs +	6 (5.7)
anti HBc IgG + (alone)	1 (0.9)

Categorical variables are reported as number (percentage). HAV: hepatitis A virus. IgG: immunoglobulin G. HBs: antibody against hepatitis B surface antigen. HBsAG: hepatitis B surface antigen. HBc: antibody against core antigen.

### Vaccine recommendations and referral to the RCSI

All patients with incomplete vaccination records were advised to seek vaccination update through the CRIE network. After identification of missing vaccine doses, the following vaccines were recommended: hepatitis A vaccine for 26 patients (25%), hepatitis B vaccine for 34 (32.7%), pneumococcal vaccine PPSV23 for 78 (75%), meningococcal conjugate vaccine for 95 (91.3%), and influenza vaccine for 48 (46.2%).

Additional vaccines included in the Brazilian National Immunization Program were also recommended, including yellow fever vaccine for 21 patients (20.2%), tetanus-diphtheria (Td) vaccine for 41 (39.4%), and MMR vaccine for 9 (8.7%).

Among the recommended vaccines, five patients (4.8%) declined yellow fever vaccination and eight (7.69%) refused COVID-19 vaccination because of concerns regarding adverse effects.

**FIGURE 1:**
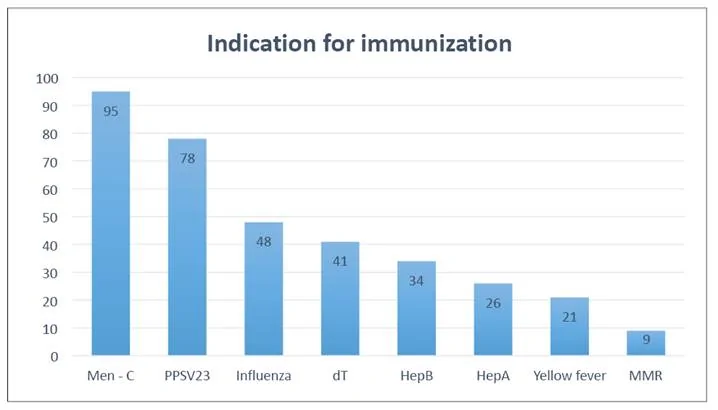
Vaccine indication and referral to the Reference Centers for Special Immunobiological for refresher vaccination.

Figure 1 illustrates the vaccine recommendations.

Men-C: Meningocócica C. PPSV23: Vacina Pneumocócica Polissacarídica 23-valente. dT: diphtheria and tetanus. HepB: hepatitis B. HepA: hepatitis A. MMR: mumps, measles and rubella.

## DISCUSSION

This study evaluated vaccination coverage among patients with chronic liver disease (CLD) followed at a tertiary referral center in Brazil and demonstrated suboptimal vaccination rates for several recommended immunobiological agents.

Patients with CLD are at increased risk of infectious complications because of progressive immune dysfunction associated with liver disease. Vaccination therefore represents an important preventive strategy, particularly in individuals with advanced fibrosis or cirrhosis.^9^ ^,^ ^11^

Despite the availability of vaccines through the Brazilian public health system, important gaps in vaccination coverage were identified in this study. These findings suggest that implementation of vaccination strategies in patients with chronic liver disease remains suboptimal in routine clinical practice. Coverage rates for pneumococcal, meningococcal, hepatitis B, and influenza vaccines remained lower than expected, even in a specialized hepatology center.

A high prevalence of positive anti-HAV IgG serology was observed. However, the low frequency of documented hepatitis A vaccination suggests that natural exposure or undocumented previous vaccination may explain most cases of seropositivity. This finding may also be related to the age profile of the participants and the epidemiological characteristics of some Brazilian regions^12^.

Regarding hepatitis B, a proportion of patients remained susceptible despite formal vaccination recommendations. Previous studies have demonstrated that vaccine response may be reduced in patients with advanced liver disease, reinforcing the importance of early immunization^13^ ^,^ ^14^ ^,^ ^15^.

Influenza and pneumococcal vaccination coverage also remained below ideal levels. These findings are clinically relevant because respiratory infections are associated with increased morbidity and mortality in cirrhotic patients^3^ ^,^ ^7^.

Meningococcal vaccination showed the lowest coverage rate among the vaccines specifically recommended for patients with CLD. Although evidence regarding meningococcal vaccination in CLD remains limited, current international recommendations support vaccination in selected patients with chronic liver disease^13^.

The present study also identified that many participants reported not receiving previous counseling regarding vaccination. However, because this information was based on patient self-report, no direct conclusions regarding physician knowledge or professional awareness can be established. Nevertheless, these findings may suggest missed opportunities for vaccination counseling in clinical practice.

This study has important limitations. First, its cross-sectional design precludes causal inferences regarding factors associated with vaccination status. Second, the use of a convenience sample from a single tertiary referral center may limit generalizability and introduce selection bias. Third, reliance on vaccination cards and self-reported information may have resulted in recall bias and possible misclassification of immunization status. Additionally, the relatively low educational level observed in part of the study population may have influenced vaccination adherence and maintenance of vaccination records. Finally, the predominance of hepatitis C in the study population may not reflect the current etiological distribution of CLD in other settings.

## CONCLUSION

Vaccination coverage among patients with chronic liver disease remained lower than expected for several recommended vaccines, even in a tertiary referral center.

These findings reinforce the importance of systematic vaccination assessment, maintenance of accurate vaccination records, and continued educational initiatives addressing immunization in patients with chronic liver disease.

Further multicenter studies are needed to better characterize barriers to vaccination and strategies to improve immunization coverage in this population.

## Data Availability

Data-available-upon-request
